# The Effect of Exposure to Low Levels of Chlorine Gas on the Pulmonary Function and Symptoms in a Chloralkali Unit

**Published:** 2011-02-17

**Authors:** Masoud Neghab, Fatemeh Amiri, Esmaeel Soleimani, Seyed Younus Hosseini

**Affiliations:** ^a^ Research Center for Health Sciences, Shiraz University of Medical Sciences, Shiraz, Iran; ^b^ Student Research Committee, Shiraz University of Medical Sciences, Shiraz, Iran

**Keywords:** Chlorine, Chloralkali Plant, Respiratory Function Tests, Respiratory Symptoms

## Abstract

**Background:** The present study was undertaken to ascertain whether (or not) long term occupational exposure to low (sub-TLV levels) atmospheric concentrations of chlorine gas was associated with any significant decrements in the parameters of pulmonary function and/or increased prevalence of respiratory symptoms.

**Methods:** In this retrospective cohort study that was performed in 2012, 54 workers of a local chloralkali unit and 38 non-exposed office staff were enrolled and compared. Atmospheric concentrations of chlorine gas were measured by numerous sampling with gas detector tubes. Data on respiratory symptoms were gathered using a standard questionnaire. Furthermore, spirometry test was performed for subjects both prior to and at the end of shift.

**Results:** Mean atmospheric concentration of chlorine gas was 0.27 ±0.05 ppm that was lower than the existing TLV value for this toxic irritant gas. Symptoms of respiratory diseases were significantly more frequent among exposed subjects than in referent individuals. Additionally, mean values of most parameters of pulmonary function including FEV1 (*P*=0.031), FEV1/FVC ratio (*P*=0.003) and PEF (*P*=0.005) were significantly lower than their corresponding values for unexposed subjects. Additional cross shift decrements were also noted in some lung functional capacities, although changes were not statistically significant.

**Conclusions:** Exposure to sub-TLV levels of chlorine gas is associated with statistically significant decrements in the parameters of pulmonary function as well as increased prevalence of respiratory symptoms.

## Introduction

Chlorine is a reactive gas, widely used over the last few decades in different industries. Exposure to it commonly occurs in mixing household chemicals, swimming pools, and industrial settings. Occupational exposures can occur during production, storage, and transportation of chlorine, in the manufacturing and processing of many products including plastics, rubbers, metals, papers, pharmaceuticals, pesticides, cosmetics, disinfectants, batteries, antifreeze, and adhesives. Under normal operating conditions, workers may be exposed to low levels of chlorine. However, high-level exposures can also occur if a large amount of chlorine is released during an accident ^1^. The available toxicological information about chlorine is almost entirely on its respiratory effects^2^.

The respiratory effects of occupational exposure to high concentrations of chlorine gas have been investigated ^2-4^. Poisoning symptoms, ranging from simple respiratory irritations, bronchial spasm, bronchial and alveolar damage, to pulmonary diseases, have been described and are similar in both humans and animals. Although these effects can be diminished or resolved with cessation of exposure and medical management, there is doubt as to whether long term exposure to low concentrations of chlorine gas following an acute episode can result in permanent and irreversible changes in lung functional capacities^2^.

Respiratory effects of long-term exposure to low levels of chlorine gas have not been well documented, and are subject to debate and controversy. For instance, shortness of breath, cardiac arrhythmias, chest pain, reactive airways dysfunction syndrome (RADS), tooth erosion and increased number of common cold cases have been reported because of exposure to low levels of chlorine gas. Additionally, exposure to low levels of chlorine gas has been an important factor in developing occupational asthma^2^.

Similarly, a high prevalence of pulmonary disorders in female cleaners due to exposure to chlorine-containing bleaches (0-0.4ppm) is reported (5). It is found a significant relationship between development of asthma and chronic bronchitis and exposure to this chemical^5^.

Moreover, exposure to sub-TLV levels of chlorine by swimmers during training is associated with the development of asthma and airway hyper responsiveness symptoms^6^. Conversely, Patil et al. did not find any association between occupational exposure to 0.15 ppm of chlorine gas for 10.9 yr and the prevalence of respiratory symptoms such as dyspnea, increased cases of common old, cardiac arrhythmias and chest pain in chloralkali workers. No evidence of permanent lung disorders was found during clinical examinations, radiography and spirometry of these workers^7^. No clinical significant symptoms of respiratory disorders in workers exposed to chlorine gas for 10 yr at concentrations twice as high as Patil’s study (0.298 ppm) was found ^8^.

Therefore, controversy exists considering the potential of chlorine gas to induce respiratory symptoms and/or lung functional impairments, particularly, at low exposure levels. Hence, the present study was, undertaken to address further this issue.

## Methods

This retrospective cohort study was performed at a chloralkali unit of a local petrochemical complex in 2012, where chlorine gas and caustic soda are produced in a process called mercury cell process^9^.

In brief, in the chloralkali electrolysis process, an aqueous solution of sodium chloride is electrolyzed electrolytically by direct current, producing chlorine, hydrogen, and sodium hydroxide solution. The overall reaction of the process takes place in two parts, at the anode and at the cathode. There are three basic processes for the electrolytic production of chlorine, the nature of the cathode reaction depending on the specific process as follows: (1) the diaphragm cell process,(2) the mercury cell process and (3) the membrane cell process. In the mercury cell process, sodium amalgam is produced at the cathode. The amalgam reacts with water in a separate reactor, called the decomposer, to produce hydrogen gas and caustic soda solution. Because the brine is recirculated, solid salt is required for resaturation. The brine, which must be quite pure, is first dechlorinated and then purified by a straightforward precipitation – filtration process. The products are extremely pure. The chlorine, along with a little oxygen, generally can be used without further purification. The sodium hydroxide solution contains little chloride and leaves the decomposer with a 50wt% concentration^10^.

Fifty four subjects working in a local chloralkali unit were studied. Similarly, 38 non-exposed subjects whose age, sex, work experience, smoking habits, socioeconomic status, level of education, and place of living were in parallel with the exposed subjects; were randomly selected from office staff of the plant as referent group. None of the exposed subjects had history of exposure to chemicals known to induce pulmonotoxicity. Additionally, they were free of preexisting medical conditions, particularly those of respiratory illnesses, chest operations or injuries. Likewise, none of the subjects in the referent group had been exposed occupationally or non-occupationally to chlorine or other chemicals known to cause respiratory symptoms or ventilatory disorders.

All subjects signed an informed consent form before participating in the study. The study was approved by the Ethics Committee of Shiraz University of Medical Sciences. The study was conducted in accordance with the Helsinki Declaration of 1964 as revised in 2013^11^.

Subjects were interviewed and respiratory symptom questionnaire, as suggested by the American Thoracic Society^12^ were filled out for them. The questionnaire contained questions concerning respiratory symptoms such as cough, wheezing and dyspnea, smoking habits, occupational history, preexisting medical conditions and family history of each employee.

The prevalence of symptoms of respiratory diseases was then calculated from the data extracted from the completed questionnaires. Information extracted from the questionnaires was then used to obtain symptom prevalence data among the exposed and unexposed groups.

Pulmonary function test (PFT) was performed according to the guidelines given by the ATS^13^ and measured with a portable calibrated vitalograph spirometer (Vitalograph-COMPACT, Buckingham-England) on site, twice for the exposed subjects (at the beginning and at the end of shift) and once for the referent individuals, details of which have been discussed elsewhere^14,15^.

Atmospheric concentrations of chlorine gas were estimated using gas detector tubes. Colorimetric tubes are regarded as +/-35 percent accurate with measurements down to one-half the exposure limit and +/-25 percent accurate up to five times the exposure limit^16^.Thirty samples were taken during four different shifts (Saturday morning and afternoon and Monday morning and afternoon) in different parts of the plant, which was a relatively small workshop.

Data were analyzed using INSTAT software and SPSS software version 20 (Chicago, IL, USA). The independent student’s *t*-test and chi-square tests were used. A *P* value of <0.05 was considered statistically significant.

Moreover, both simple logistic regression and multiple logistic regression analyses were performed to analyze the association between exposure to chlorine gas and symptoms of respiratory diseases. Smoking status was considered as a potential confounder in the model.

## Results

Demographic variables, smoking habits and area concentrations of chlorine are presented in Table 1. Only length of employment (*P*=0.015) and length of smoking of referent subjects (*P*=0.021) were significantly higher than those of exposed subjects. The mean duration of exposure for exposed group was 6.76±6.86 yr, the number of smokers among exposed and referent subjects were 17 and 10, respectively and the difference was not statistically significant. Chlorine concentrations showed little variability in different areas (about 0.2 ppm). Workers did not wear any respiratory protective equipment during their daily activities over the course of their employment.

**Table 1:** Demographic characteristics of exposed and unexposed subjects

**Parameter**	**Exposed** **n=54**		**Unexposed** **n=38**		***P*** ** value**
	**Mean**	**SD**	**Mean**	**SD**	
Age (yr)	34.83	8.47	36.10	6.87	0.224
Weight (kg)	73.64	9.11	73.78	10.97	0.474
Height (cm)	173.98	6.37	172.23	5.70	0.089
Duration of employment (yr)	6.76	6.86	10.94	9.32	0.015
Length of smoking (yr)	5.28	3.79	7.00	4.10	0.021
Chlorine concentration (ppm)	0.27	0.05	0.00	0.00	-

Table 2 illustrates the prevalence of respiratory symptoms. As shown, frequency of cough, phlegm, productive cough and wheezing were significantly higher in exposed subjects than in referent group.

**Table 2:** Prevalence of respiratory symptoms in exposed and unexposed subjects

**Variables**	**Exposed**	**Unexposed**	**Unadjusted OR (95% CI)**	***P*** ** value**	**Adjusted OR (95% CI)** ^a^	***P*** ** value**
Cough	30	2	22.50 (4.91, 103.05)	0.001	22.74 (4.92, 105.05)	0.001
Phlegm	36	4	17.00 (5.22, 55.35)	0.001	18.91 (5.49, 65.14)	0.001
Productive cough	13	1	11.73 (1.46, 94.09)	0.020	11.56 (1.44, 93.17)	0.021
Wheezing	12	1	10.57 (1.31, 85.24)	0.027	10.41 (1.28, 84.12)	0.028
Breathlessness	26	11	2.27 (0.94, 5.50)	0.067	2.33 (0.91, 5.96)	0.079

^c^ adjusted for smoking

Univariate analysis (unadjusted OR) revealed that the prevalence of cough (*P*=0.001), phlegm (*P*=0.001), productive cough (*P*=0.020) and wheezing (*P*=0.027) were significantly higher in exposed group than in referent individuals. When the potential confounding effects of smoking was controlled (adjusted OR in multiple logistic) the association between exposure to chlorine gas and the prevalence of cough (*P*=0.001), phlegm (*P*=0.001), productive cough (*P*=0.021) and wheezing (*P*=0.028) remained significant.

Table 3 depicts the results of spirometry. As shown, FEV_1_ (*P*=0.031), FEV_1_/FVC ratio (*P*=0.003) and PEF (*P*=0.005) were significantly lower in exposed group than in referent individuals. All parameters of pulmonary function showed additional decrements at the end of shift, although the difference did not reach statistical significance.

**Table 3:** Percentage predicted pulmonary function among exposed and unexposed subjects

**Parameter**	**Exposed, n=54**								
	**Before shift**	**After shift**	**Unexposed, n=38**		***P*** ** value**			
	**Mean**	**SD**	**Mean**	**SD**	**Mean**	**SD**	**Before vs. After**	**Before vs. Unexposed**	**After vs. Unexposed**
FVC	91.97	9.62	91.44	11.35	94.05	13.61	0.397	0.196	0.160
FEV1	86.13	13.25	82.75	9.91	91.39	13.11	0.068	0.031	0.001
FEV1/FVC	92.07	9.88	90.29	8.64	97.23	6.94	0.161	0.003	0.001
PEF	79.79	21.48	75.35	17.98	90.81	17.16	0.123	0.005	0.001

## Discussion

The present study examined chronic and acute respiratory effects of exposure to sub-TLV levels of chlorine gas. Exposed subjects were free from preexisting medical conditions. Additionally, there were no significant differences between both groups as far as major confounding variables were concerned.

The mean concentration of chlorine gas was 0.27 ±0.05 ppm, which is lower than the existing TLV value of 0.5 ppm recommended by ACGIH ^17^ for this toxic, irritant gas. This small variability indirectly indicates that the mean value reported here would be an acceptable predictor of overall exposure scenario.

Our findings indicate that the prevalence of respiratory symptoms was significantly higher in exposed subjects than in the non-exposed employees.

Similar findings have been reported by some other investigators. Kennedy et al. compared 321 pulp mill workers with a control group of 237 rail yard workers in similar working conditions but not exposed to chlorine. Average chlorine levels in the pulp mill were below 1 ppm. Pulp mill workers (both smokers and nonsmokers) who reported being gassed were significantly more likely to report wheezing on occasion than were other pulp mill workers and rail yard workers^18^. Similarly, Enarson et al. compared 392 pulp mill workers exposed to chlorine with a group of 310 rail yard workers living in the same community, but not exposed to chlorine. Workers were exposed to an average of 0.02 ppm of Cl_2_. They reported increased prevalence of chest tightness and wheezing in workers exposed to chlorine gas at a pulp mill industry^19^. Interestingly, different results have also been reported by others. For instance, Rotman et al. studied the effects of exposure to low levels of chlorine (0.5 or 1.0 ppm) on normal volunteers and found that even though chlorine at low concentrations did not produce any serious subjective symptoms, it adversely affected pulmonary function transiently^20^. Likewise, Chasis et al. did not find any evidence of chlorine-induced pulmonary disease in 29 hospitalized cases exposed to low levels of chlorine gas^21^.

Mean values of most, before shift and after shift, parameters of pulmonary function of exposed subjects were significantly lower than those of referent subjects were. Additionally, all parameters of pulmonary function showed additional decrements at the end of shift, although, the difference did not reach statistical significance.

In the line with these observations, Boskabady et al. studied the effects of exposure to chlorine on pulmonary function tests in Iranian lifeguards and showed that exposure to chlorine was associated with significant reductions in PFT values of lifeguards^22^. Similarly, McCord studied industrial poisoning from low concentrations of chlorine gas and reported that the worker might have had a decrease in pulmonary function because of chlorine exposure^23^. Surprisingly, discrepant results on chlorine exposure and PFT changes have also been reported. Ferris et al. compared 147 men in a pulp mill industry to 124 men in a paper mill. The former had previous exposure to chlorine and sulphur dioxide gases. No significant differences were noted in the results of PFTs between both groups. The prevalence of respiratory symptoms in both plants was lower than that of the male population in the community^24^. Patil et al. studied the health of Diaphragm Cell Workers Exposed to Chlorine. Level of occupational exposure to chlorine gas was 0.146±0.287ppm. No evidence of permanent lung disorders was found during clinical examinations, radiography and spirometry of these workers^7^.

Although the exact reason(s) of these inconsistencies and discrepancies are not known, differences in exposure concentrations, length of exposure, occupational and non-occupational exposure to other toxic agents in the past, present co-exposure to other chemicals, the possibility of exposure to higher or lower concentrations of chlorine gas in the past, use of respiratory protective equipment, and a difference in sample size may explain, at least in part, these discrepancies.

To differentiate between acute and chronic effects of chlorine, PFTs were measured twice for the exposed subjects, first, after a 2-day exposure free period (prior to shift) and the second at the end of shift.

Apart from FVC, the pre-shift (obtained after a 48 h exposure free period) values of all parameters of pulmonary function of exposed subjects were significantly lower than those of referent subjects, indicating that exposure to chlorine has been associated with chronic irreversible decrements in some parameters of pulmonary function.

This observation was made while unexposed employees were, on average, more than a year older than exposed employees were, had worked about 4 yr more than the exposed subjects work and had smoked about two years more than their exposed counterparts had.

Although additional post-shift decrements were noted in PFTs of exposed subjects, their difference with pre-shift values of the same individuals did not reach statistical significance, indicating that under the existing scenario, exposure to chlorine was not associated with acute changes in PFTs.

It may be argued that the mean values of most parameters of pulmonary function have exceeded 80%, and therefore, this could be viewed as a normal spirogram result^25^. However, over 10% difference between the results of spirometry of exposed and unexposed individuals and about 5% decrement in the parameters of pulmonary function of exposed subjects at the end of shift, both of which being statistically significant, could not simply be overlooked. In fact, it is important to note that OSHA considers FEV1 declines of as little as 5% to be clinically significant^26^.

Given the above and in view of the pattern of the spirometry results (significant decrements in FEV1 and FEV1/FVC ratio of exposed employees^27^), it would be plausible to assume that long term consequences and ramifications of these changes are likely to result in obstructive pulmonary disease, consistent with the mechanism of effect of chlorine.This proposition is also in line with the previous findings, which showed abnormal radiographic signs (diffuse bronchiolitis) and abnormal PFTs (obstructive ventilatory disorders)^18,28^.

Due to the inherent limitations of the retrospective cohort studies, cause and effect relationships could not be established from studies such as the present investigation. Therefore, one might argue that observed changes are not necessarily related to exposure to chlorine. While the authors agree with this view, it should be noted that a few lines of circumstantial evidence support the proposition that the observed abnormalities are likely to be the direct consequence of exposure to chlorine.

First, none of the exposed subjects had any history of respiratory diseases or preexisting medical conditions at the beginning of their employment in the plant or prior to it. Second, exposed subjects did not have any history of exposure to any other chemicals known to cause respiratory disorders, during the course of their employment. Third, despite the fact that referent subjects were older than their exposed counterparts and their length of employment and years smoked were significantly higher than those of exposed individuals, the prevalence of respiratory symptoms and lung functional disorders were significantly higher in exposed workers. Fourth, although PFT values showed a relative recovery after a 48-h exposure free period, they still were significantly lower than those of referent subjects were. Fifth, when the effects of an important potential confounder, cigarette smoking, was controlled, significant association remained between exposure to chlorine gas and the prevalence of respiratory symptoms.

Additional, longitudinal, follow up cohort studies with larger sample sizes are clearly needed to further substantiate these preliminary findings in group of workers whose exposure to chlorine have been quantified by more sensitive methods than the semi-quantitative method used in this study due to technical difficulties and lack of equipment in our laboratory.

## Conclusions

Long-term occupational exposure to even sub-TLV concentrations of chlorine gas can be a risk factor for chronic lung disorders manifested by significant increases in the prevalence of respiratory symptoms and subtle chronic irreversible decrements in the parameters of pulmonary function. Thus, engineering control measures and the use of appropriate respirators are recommended to eliminate or reduce workers’ exposure to chlorine. Active rather than common periodic examinations are recommended to identify susceptible workers prior to developing permanent irreversible respiratory disorders.

## Acknowledgments

Funding through the Shiraz University of Medical Sciences, Vice Chancellor for Research and Technology, contract no. 86-3456, partially supported this study. The authors also wish to thank Mr Moayedi for his technical assistance.

## Conflict of interest statement

The authors have no conflict of interest to declare.
